# TLR9 polymorphism correlates with immune activation, CD4 decline and plasma IP10 levels in HIV patients

**DOI:** 10.1186/s12879-019-3697-9

**Published:** 2019-01-16

**Authors:** Anjali Joshi, Erin B. Punke, Tugba Mehmetoglu-Gurbuz, Diego P. Peralta, Himanshu Garg

**Affiliations:** 1 Department of Biomedical Sciences, Center of Emphasis in Infectious Diseases, Texas Tech University Health Sciences Center, 5001 El Paso Dr, El Paso, TX 79905 USA; 2 Division of Infectious Diseases, Department of Internal Medicine, Texas Tech University Health Sciences Center, El Paso, TX 79905 USA

**Keywords:** HIV, AIDS, TLR9 1635A/G, TLR9 1486C/T, IP10, TLR9 polymorphisms, Immune activation, Plasma biomarkers

## Abstract

**Background:**

The mechanism behind HIV mediated immune activation remains debated, although the role of virus replication in this process is increasingly evident. Toll like Receptor 9 (TLR9) has been implicated in HIV mediated immune activation via sensing of viral CpG DNA. Polymorphisms in the TLR9 gene and promoter region including TLR9 1635A/G and 1486C/T have been found to be associated with multiple infectious diseases and cancers.

**Methods:**

In the current study, we looked at the correlation of TLR9 polymorphisms 1635A/G and 1486C/T with key hallmarks of HIV disease in a cohort of 50 HIV infected patients. We analyzed CD4 counts, T cell immune activation characterized by upregulation of CD38 and HLA-DR and upregulation of plasma biomarkers of inflammation like LPS, sCD14, IL-6 and IP10 in the HIV patient cohort and compared it to healthy controls.

**Results:**

We found that TLR9 1635AA genotype was associated with lower CD4 counts and significantly higher immune activation in both CD4+ and CD8+ T cells. Analysis of HIV associated plasma biomarkers including LPS, sCD14, IL-6 and IP10 revealed a strong correlation between IP10 and immune activation. Interestingly, IP10 levels were also found to be higher in HIV patients with the 1635AA genotype. Furthermore, the TLR9 1486C/T polymorphism that is in linkage disequilibrium with 1635A/G was weakly associated with lower CD4 counts, higher CD8 immune activation and higher IP10 levels.

**Conclusions:**

As TLR9 stimulation is known to induce IP10 production by dendritic cells, our findings provide new insights into HIV mediated immune activation and CD4 loss. TLR9 stimulation by viral CpG DNA may be important to HIV immunopathogenesis and the TLR9 polymorphisms 1635A/G and 1486C/T may be associated with disease progression.

## Background

Immune activation remains a hallmark of HIV disease and correlates with multiple pathological features like CD4 loss, disease progression and viremia [1, 2]. In HIV infections, different markers of immune activation are upregulated in T cells, B cells, natural killer cells and dendritic cells in the peripheral blood along with upregulation of inflammatory cytokines both in treatment naïve and ART experienced patients [3–5]. Different mechanisms have been proposed for the immune activation seen in HIV infections including microbial translocation via the damaged gut [6], imbalance in cytokine production [7], chronic elevation in interferon levels [8], direct effect of virus replication, etc. [9]. The involvement of virus replication in immune activation is supported by several findings including higher immune activation seen in viremic patients [2], reduction in immune activation after initiation of ART [4, 10–12] and association of immune activation with residual viremia in patients unable to suppress virus replication [13].

The direct role of viremia in HIV disease raises the possibility that innate immune sensors may play a key role in HIV mediated immune activation [14, 15]. During infection by a micro-organism, Pattern Recognition Receptors (PRRs) like TLRs expressed by the cells of the innate immune system initiate an immune response which is characterized by localized inflammation, cytokine secretion and recruitment of effector cells. As TLR expression varies by cell type and tissue, and as TLR activation involves many adaptor and signaling proteins, different pathogens may activate TLRs differently. TLRs interact with adaptor proteins via their cytoplasmic domains to activate transcription factors with a general outcome of type I interferon (IFN) production and secretion of pro-inflammatory cytokines [16].

To date, several TLRs have been implicated in HIV replication, disease progression and pathogenesis [14, 17–19] and blocking the TLR signaling pathway has been proposed as an immunomodulant in HIV infected patients [20]. TLR9 is expressed by a variety of immune cells like macrophages, dendritic cells, PBMCs etc. and recognizes unmethylated CpG DNA motifs found in viruses and bacteria. Interestingly, signaling via TLR9 has been proposed as one of the mechanisms behind HIV induced immune activation by stimulating plasmacytoid dendritic cells to secrete interferon alpha (IFN-α) [14, 15, 17, 21]. Furthermore, TLR ligands have been shown to induce immune activation in vitro in CD4 and CD8 T cells derived from HIV patients [19] that may contribute to immune dysfunction and HIV pathogenesis.

The remarkable variability in the clinical course of HIV infection can partially be explained by differences in the host genetic make-up and gene polymorphisms [22]. Host specific differences in the innate immune system like TLR9 gene are likely to alter the course of an adaptive immune response, especially in a chronic infection like HIV [23, 24]. Different polymorphisms in the TLR9 gene have been described and known to be associated with diseases like bacterial meningitis, CMV infection, toxoplasmosis, malaria and SLE [25–29]. Of those, the TLR9 1635A/G polymorphism (rs352140), is most widely studied and shown to be significantly associated with several infectious diseases [26, 28, 30, 31]. TLR9 1635A/G polymorphism has also been previously studied in context of HIV infection and associates with HIV acquisition/infection [30, 32], disease progression [33, 34], CD4 counts [35, 36] and viral load [37]. While the presence of 1635AA has been associated with increased risk of HIV acquisition, lower CD4 counts and higher viral loads [30, 36–38], paradoxically, 1635AA was also associated with lower viral loads combined with slower disease progression [33, 35] in other studies. It is possible that these varying outcomes between studies may be due to genetic differences between the study populations. However, the mechanism via which this polymorphism influences HIV disease parameters remains unknown. Another TLR9 polymorphism in the promoter region, 1486C/T (rs187084) has been associated with diseases like SLE [39], rheumatoid arthritis [40], HPV infection, pulmonary tuberculosis [41] and certain cancers [42]. Interestingly, there are no studies linking this polymorphism to HIV disease.

In the current study, we looked at the correlation of TLR9 polymorphisms 1635A/G and 1486C/T with key hallmarks of HIV disease like CD4 counts, T cell immune activation and upregulation of plasma biomarkers of inflammation. We found that TLR9 1635AA and 1486CC genotypes, either alone or in combination, were associated with lower CD4 counts, significantly higher T cell activation or elevated IP10 levels in HIV patients. These findings shed fundamental insights into the role of host TLR9 genotype in HIV disease, especially by modulating immune activation.

## Methods

### Ethics, consent and permissions

The study was reviewed and approved by the Texas Tech University Health Sciences Center’s regional Institutional Review Board (IRB). All methods were performed in accordance with the relevant guidelines and regulations. The study design was cross-sectional and the study number recorded as IRB# E12092, approval date 07/31/2012. All participants were provided with written and oral information about the study. Written informed consent of all study participants in accordance with the Institutional policy was documented. All participants were identified by coded numbers to assure anonymity and all patient records kept confidential.

### Patient population

Fifty HIV-infected individuals and 29 healthy controls were recruited from the outpatient HIV clinic at the Texas Tech University Health Sciences Center at El Paso**.** The mean age of the HIV+ patient population was 37.9 ± 11.9. The HIV group comprised of 9 females (18.0%) and 41 males (82.0%). The study was cross sectional consisting of patients at different stages of the disease. An age matched HIV- population control group (*n* = 29) was also recruited from the same geographical location and comprised of 10 females (34%) and 19 males (65.5%). The mean age of the healthy control group was 34.7 ± 9.8.

### Sample collection and storage

Each patient provided a 20 ml blood sample that was separated into plasma and cellular components using Ficoll based separation. Genomic DNA was extracted from whole blood samples using the QIAamp DNA Blood Mini kit (Qiagen). All components from the sample including plasma, cells and DNA were aliquoted and stored at − 70 °C till further analysis.

### TLR9 polymorphisms

PCR-RFLP was performed to determine the TLR9 1635A/G polymorphism in the TLR9 gene at position 1635 to determine genotypes of all patient samples. For this, the TLR9 gene was PCR amplified surrounding position 1635 (nucleotide bases 1219–1830, numbering from the ORF of the TLR9 gene) using forward primer 5’-CAG CTC GGC ATC TTC AGG GCC TTC-3′ and reverse primer 5’-CAG TGC ATT GCC GCT GAA GTC CAG-3′. The resulting PCR product was digested with BstUI enzyme resulting in 2 bands at 417 and 195 bp for the homozygous GG genotype; one band at 612 bp for homozygous AA and three bands at 612, 417, and 195 bp for heterozygous GA. For TLR9 1486 C/T polymorphism, the TLR 9 gene surrounding the position 1486 was PCR amplified using the forward primer 5′ - CTA TGG AGC CTG CCT GCC ATG ATA CC - 3′ and the reverse primer 5′ - CTG GTC ACA TTC AGC CCC TAG AG - 3′. The resulting 755 bp PCR fragment was digested with AflII resulting in one band at 755 bp for homozygous CC, three bands at 755, 505 and 250 bp for heterozygous CT and two bands at 505 and 250 bp for homozygous TT.

### Immunostaining

Staining of cells for different immune markers has been described previously [2, 43]. Briefly, lymphocytes isolated from the blood samples obtained from HIV-infected or normal patients were stained for cell surface markers using specific antibodies: CD3-Cy7, CD4-Tx red, CD8-APC (Beckman Coulter), CD38 PE, HLA-DR FITC, CCR5 PE (BD Pharmingen) and CaspACE FITC-VAD-FMK (Promega). Stained cells were fixed using the Cytofix reagent (Beckman Coulter) and run on a 10 color Beckman Coulter Gallios Flow Cytometer. At least 20,000 events for each sample were acquired. Data was analyzed using the FlowJo software (Tree Star). Cells were first gated on the CD3+ population and CD4+ and CD8+ T cell subsets determined.

### IL-6, IP10, sCD14 and LPS determination

IL-6, IP10 and sCD14 levels in the plasma samples from HIV infected patients and healthy controls were determined via specific ELISAs. For IL-6 determination, the Quantikine HS ELISA kit (R&D Systems) was used following the manufacturer’s protocol. The kit is designed to measure human IL-6 in serum, plasma and urine and has a sensitivity of 0.016–0.110 pg/mL. IP10 and sCD14 levels were determined using the Quantikine ELISA human IP10 and CD14 Immunoassay (R & D Systems) following the manufacturer’s protocol. The sensitivity of IP10 detection using the kit is 0.41–4.46 pg/mL and the minimum detectable dose (MDD) of human CD14 is less than 125 pg/mL. Plasma LPS levels were determined using the endpoint chromogenic Limulus Amebocyte Lysate (LAL) assay (Lonza) using the manufacturer’s recommendations. The kit shows linear absorbance at 405–410 nm in the concentration range of 0.1–1.0EU/mL endotoxin.

### Statistical analysis

Data were analyzed using GraphPad Prism (GraphPad Software, Inc., La Jolla, CA). Differences between groups were assessed using the students one tail t-test; and *p* values were considered significant at *p* < 0.05. Comparison between multiple groups was done using the Kruskal-Wallis test. Spearman’s correlation with linear regression was used for all correlation determination using the GraphPad Prism Software.

## Results

### TLR9 1635AA genotype is associated with lower CD4 counts in HIV positive patients

**Fig. 1:**
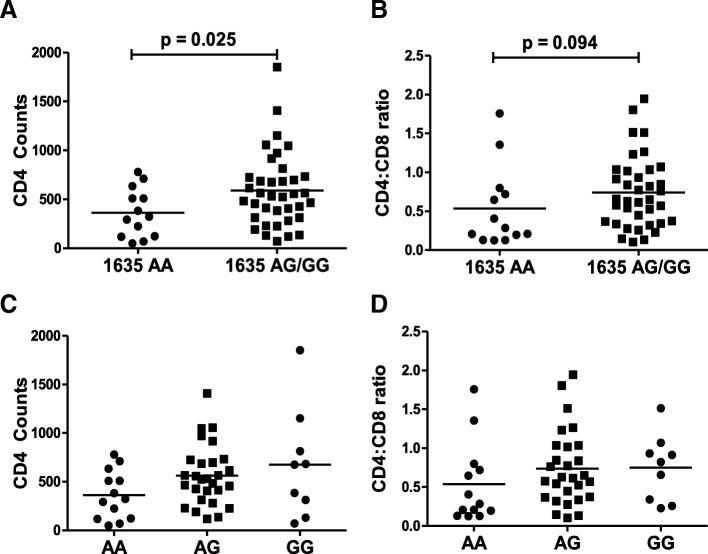
TLR9 1635AA genotype is associated with lower CD4 counts in HIV infected patients: DNA was isolated from whole blood from 50 HIV infected patients and TLR9 1635 polymorphism determined by PCR-RFLP analysis. CD4 and CD8 levels from the patient blood samples were determined by immunostaining followed by flow cytometry. TLR9 1635AA or AG/GG genotypic groups were compared for (**a**) CD4 counts and (**b**) CD4:CD8 ratio. Patient population was subdivided into TLR9 1635AA, AG or GG genotypes and (**c**) CD4 counts and (**d**) CD4:CD8 ratio were compared. One tailed student’s t-test or Kruskal-Wallis test were used for statistical analysis

Selective depletion of CD4+ T cells via apoptosis followed by reduction of CD4:CD8 ratio is a hallmark of HIV disease progression [44]. The course of HIV infection is highly variable in different individuals. This variability is manifested not only as differential rates of disease progression [45] but also in recovery after HAART administration [13]. Differences in the genetic make-up of the host like TLR polymorphisms can in part explain some of the variability in HIV disease progression [34]. We hence looked at TLR9 1635A/G polymorphism and its association with HIV clinical parameters in a cross sectional patient cohort. The frequency of TLR9 1635A/G polymorphism was determined via PCR-RFLP and distribution of this polymorphism between HIV infected and healthy controls was found to be similar (Table 1). Further analysis showed that patients with TLR9 1635AA genotype had significantly lower (*p* = 0.025) CD4 counts than subjects with the 1635GG/AG genotype **(**Fig. 1a)**.** Interestingly, although not significant, a similar trend was seen with CD4:CD8 ratio with 1635AA genotype being associated lower CD4:CD8 ratio than 1635AG/GG (Fig. 1b). These data are in agreement with previous findings [36] and indicate an association of TLR9 1635A/G polymorphism with CD4 levels in HIV infection. In order to better understand the involvement of 1635AA, AG or GG genotype in CD4 decline, we further stratified the CD4 decline data according to the respective genotypes. As demonstrated in Fig. 1c, the 1635AA genotype was associated with the lowest CD4 counts as well as CD4:CD8 ratio (Fig. 1d)**.** This was followed by intermediate CD4 counts for the AG and highest CD4 counts for the GG polymorphism both in terms of CD4 counts and the CD4:CD8 ratio. These data demonstrate that the TLR9 1635AA genotype is associated with lower CD4 counts in HIV infected individuals.

**Table 1:** Frequency of TLR9 1635A/G and 1486C/T polymorphisms in the HIV positive subjects versus HIV negative controls

	TLR 9 Polymorphism	HIV + N (%)	HIV-N (%)
1635	AA	13 (26)	8 (27.5)
	AG	28 (56)	17 (58.6)
	GG	9 (18)	4 (13.8)
1486	CC	7 (14)	5 (17.2)
	CT	30 (60)	14 (48.2)
	TT	13 (25)	10 (34.5)
1635–1486	AA-CC	6 (12)	5 (17.2)
	AA-CT	6 (12)	3 (10.3
	AA-TT	1 (2)	0 (0)
	AG-CC	1 (2)	0 (0)
	AG-CT	24 (48)	11 (37.9)
	AG-TT	3 (6)	6 (20.6)
	GG-TT	9 (18)	4 (13.8)

### TLR9 1635AA genotype is associated with higher immune activation in both CD4+ and CD8+ T cells

**Fig. 2:**
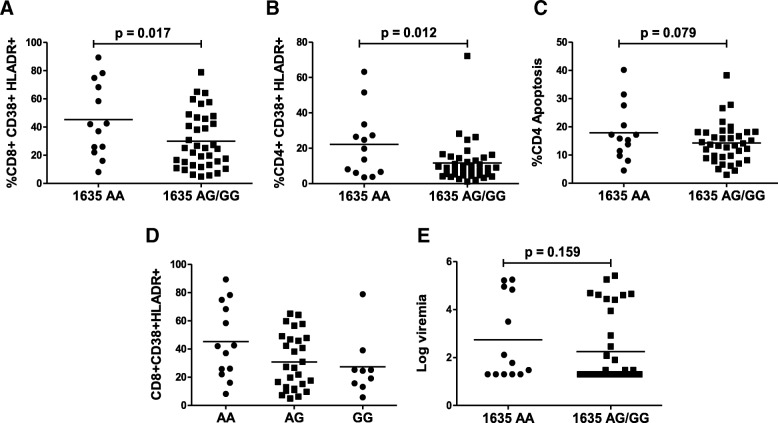
TLR9 1635AA genotype is associated with higher T cell immune activation. Immune activation was determined as CD38+ HLA-DR+ cells in CD4 and CD8 populations by flow cytometry. Immune activation in (**a**) CD8+ or (**b**) CD4+ T cells in TLR9 1635AA versus AG/GG genotype. (**c**) CD4 T cell apoptosis determined by z-VAD-FITC staining in TLR9 1635AA vs AG/GG groups. (**d**) Immune activation in CD8 T cells between 1635AA, AG or GG genotypes. (**e**) Comparison of plasma viremia between TLR9 1635AA or AG/GG genotypes. One tailed student’s t-test or Kruskal-Wallis test was used for statistical analysis

Non-specific activation of both CD4+ and CD8+ T cells characterized by up regulation of markers like CD38 and HLA-DR is a key characteristic of HIV infection [10]. Although the precise mechanism of HIV induced immune activation remains controversial, several hypotheses have been put forth to explain the phenomenon including a role of TLRs [5, 46]. We looked at the association of TLR9 1635A/G polymorphism with immune activation in CD4+ and CD8+ T cells, measured as %CD38 + HLA-DR+ cells, in each cell populations. As demonstrated in Fig. 2**,** the 1635AA genotype was associated with significantly higher immune activation in both CD8+ (*p* = 0.017) (Fig. 2a) and CD4+ (*p* = 0.012) (Fig. 2b) T cells. As HIV infection is characterized by specific depletion of CD4 T cells, we also looked at the association of TLR91635A/G polymorphism with CD4+ T cell apoptosis. As shown in Fig. 2c, although not significant (*p* = 0.079), TLR9 1635AA polymorphism showed a trend towards higher CD4+ T cell apoptosis when compared to 1635AG/GG. Further analysis of individual contributions of the TLR91635AA, AG or GG genotype in CD8 immune activation showed that the 1635AA genotype was associated with highest immune activation followed by AG and GG genotypes (Fig. 2d) consistent with the CD4 count data. However, we did not find a significant correlation of TLR9 1635A/G polymorphism with plasma viremia (Fig. 2e). This might be because not all the patients in our study population were on HAART at the time of sample collection. These data suggest that TLR9 1635A/G polymorphism may influence HIV disease by regulating immune activation in both CD4 and CD8 T cells.

### Plasma biomarkers of inflammation are elevated in HIV+ patients

**Fig. 3:**
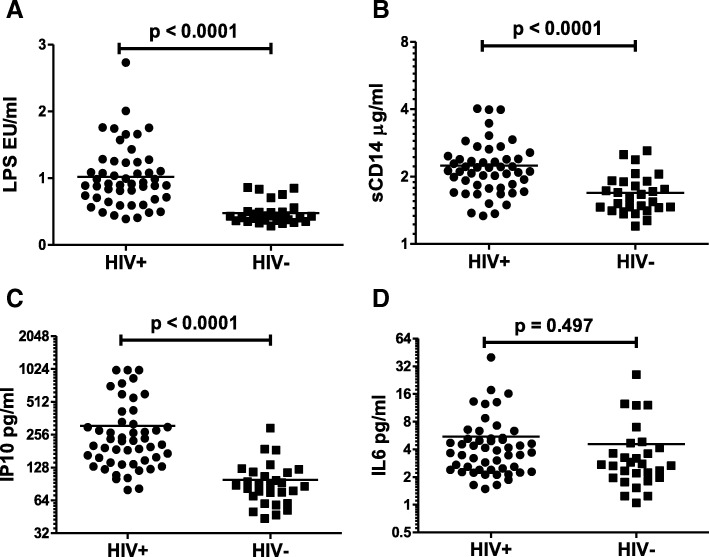
Plasma biomarkers of inflammation are elevated in HIV+ patients. Different inflammatory markers were analyzed in the plasma samples using specific ELISAs for sCD14, IP10 and IL-6 or the LAL assay for LPS. Comparison of (**a**) LPS (**b**) sCD14 (**c**) IP10 and (**d**) IL-6 levels in HIV infected individuals versus normal controls. One tailed student’s t-test was used for statistical analysis

LPS, sCD14, IL6 and IP10 are some of the plasma biomarkers that have been shown to be elevated in HIV patients [7, 47]. In fact, these inflammatory biomarkers are reliable predictors of HIV disease progression correlating consistently with CD4 decline and plasma viral load [3, 48]. To determine if these markers of inflammation were also elevated in our patient population compared to HIV- controls, we tested plasma samples for LPS using the Limulus Amebocyte Lysate (LAL) assay and quantitative ELISAs for IL6, sCD14 and IP10. Interestingly, in our patient population, LPS (Fig. 3a), sCD14 (Fig. 3b) and IP10 (Fig. 3c) levels were significantly elevated (*p* < 0.0001) when compared to HIV- controls. Although IL-6 levels were also higher in HIV+ patients compared to HIV- controls (Fig. 3d), the difference did not reach statistical significance in our study. These data confirm previous findings and emphasize the importance of nonspecific inflammatory markers in HIV disease. Furthermore, these findings demonstrate that the upregulation of inflammatory markers in our patient population are consistent with HIV disease.

### TLR9 1635AA polymorphism correlates with higher plasma IP10 levels in HIV infected patients

**Fig. 4:**
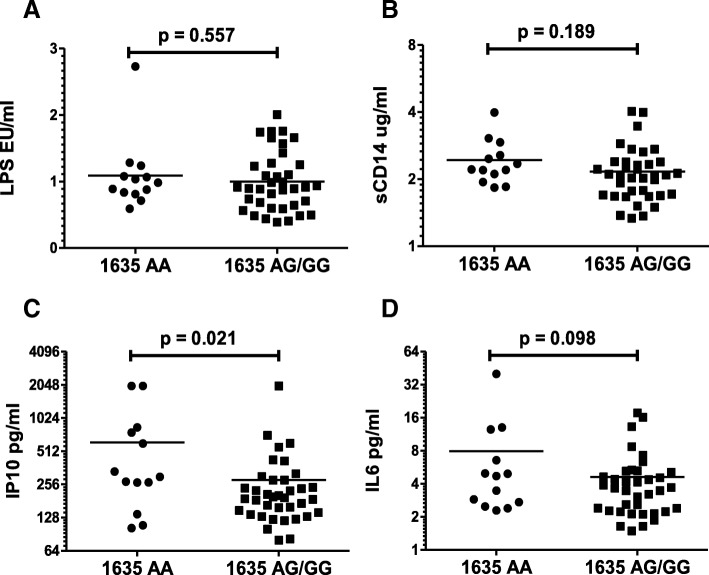
TLR9 1635AA genotype is associated with higher IP10 levels in HIV+ patients. The indicated inflammatory markers were analyzed in the plasma samples using specific ELISAs or the LAL assay for LPS. Differences between TLR9 1635AA and AG/GG genotype with respect to plasma (**a**) LPS (**b**) sCD14 (**c**) IP10 and (**d**) IL-6 levels in HIV infected individuals. One tailed student’s t-test was used for statistical analysis

Our data above showed elevated LPS, sCD14, IL-6 and IP10 levels in HIV infected patients and a significant correlation of the TLR9 1635AA polymorphism with both CD4 and CD8 immune activation. We next asked whether TLR9 1635A/G polymorphism correlated with any of the above inflammatory biomarkers. Although LPS (Fig. 4a), sCD14 (Fig. 4b), and IL-6 (Fig. 4d) levels were higher in the TLR9 1635AA genotype compared to AG/GG, these differences did not reach statistical significance. Interestingly, IP10 levels were significantly elevated (*p* = 0.021) in patients with the TLR91635AA genotype compared to AG/GG genotype (Fig. 4c). These findings are interesting because TLR9 stimulation has been shown to induce CD38 and HLA-DR upregulation [19] as well as mediate IP10 release from various cells [49, 50]. Taken together, our data suggest a role for TLR9 1635A/G polymorphism in HIV disease by altering HIV mediated immune activation.

### IP10 levels correlate with plasma viremia in HIV infected patients

**Fig. 5:**
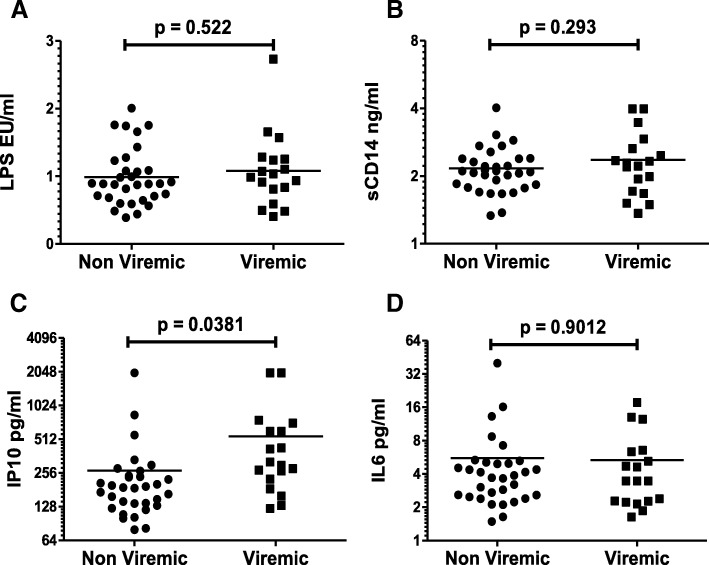
Plasma IP10 levels are higher in viremic HIV+ patients. LPS, sCD14, IP10 and IL-6 levels were determined in the plasma samples of HIV infected patients via the LAL assay or respective ELISAs. Comparison of (**a**) LPS (**b**) sCD14 (**c**) IP10 and (**d**) IL-6 levels in viremic versus non-viremic HIV infected individuals. One tailed student’s t-test was used for statistical analysis

There is direct evidence for a key role of plasma viremia in HIV induced immune activation as in most instances, markers of inflammation correlate with viral load [2] and suppressing the virus via ART also reduces immune activation [51]. We hence sought out to determine if in our HIV+ patient population, the levels of plasma biomarkers (LPS, sCD14, IP10 and IL6) were higher in viremic patients (defined as viral load > 100 copies/ml) compared to those that had their plasma viremia well controlled (≤100 copies). In our study population, LPS (Fig. 5a)**,** sCD14 (Fig. 5b), or IL6 levels (Fig. 5d), did not show a significant difference in viremic versus non-viremic patients. Interestingly, IP10 levels were significantly elevated (*p* = 0.0381) in viremic patients (Fig. 5c) compared to those that had their viremia controlled. The elevated levels of IP10 in viremic patients is consistent with a recent report showing IP10 to be a highly sensitive marker of HIV replication [52]. Moreover, these data are suggestive of a role of plasma viremia in elevating IP10 levels presumably via TLR9 signaling.

### IP10 levels correlate strongly with CD4 decline and T cell immune activation in HIV+ patients

**Fig. 6:**
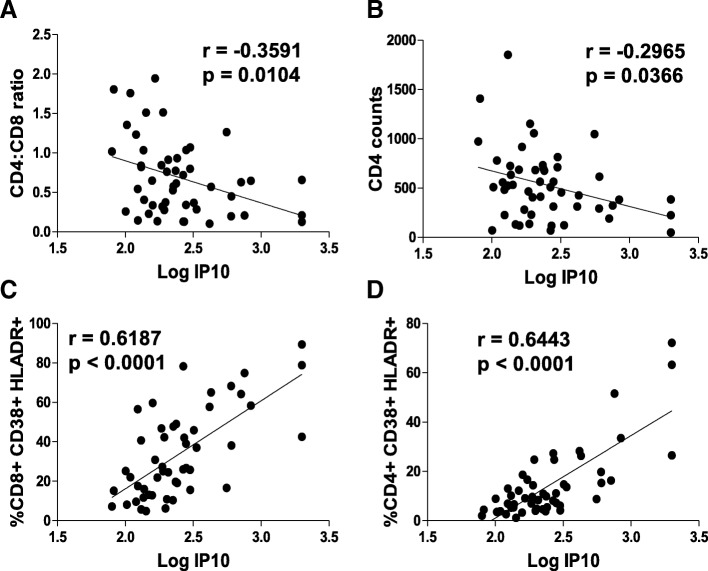
Plasma IP10 levels correlate strongly with CD4 counts, CD4:CD8 ratio and T cell immune activation in HIV infected patients. CD4, CD8 counts and T cell immune activation in the PBMCs of HIV infected patients was determined by immunostaining followed by flow cytometry. Plasma IP10 levels were determined via quantitative ELISA. Correlation analysis of plasma IP10 levels with (**a**) CD4:CD8 ratio (**b**) CD4 counts (**c**) CD8 immune activation and (**d**) CD4 immune activation in HIV infected patients. Spearman’s correlation with linear regression was used for correlation determination

Our data above showed that amongst the plasma biomarkers studied, IP10 levels correlate the strongest with viremia and immune activation. We hence conducted a correlation analysis of plasma IP10 levels with CD4 decline and T cell immune activation. Interestingly, plasma IP10 levels correlated strongly (*p* = 0.0104) with CD4:CD8 ratio (Fig. 6a), CD4 counts (*p* = 0.0366) (Fig. 6b), CD8 immune activation (*p* < 0.0001) (Fig. 6c), and CD4 immune activation (*p* < 0.0001) (Fig. 6d). This suggests that IP10 levels may be strong predictors of HIV induced CD4 decline, inversion of CD4:CD8 ratio and more importantly, CD4 and CD8 immune activation. This is consistent with the study by Pastor et al [52] where assessment of 49 inflammatory biomarkers in a cohort of HIV seronegative individuals showed that IP10 had the highest accuracy in identifying individuals with acute HIV infection.

### TLR9 1486C/T polymorphism correlates with CD4 decline, CD8 immune activation and plasma IP10 levels

**Fig. 7:**
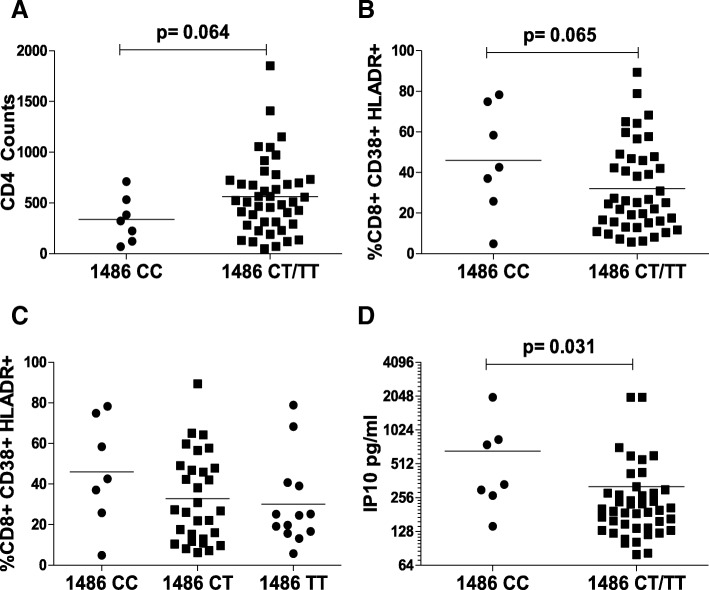
TLR9 1486CC genotype is associated with lower CD4 counts and higher CD8 immune activation and plasma IP10 levels in HIV infected individuals. TLR91486 polymorphism was determined by PCR-RFLP analysis. Plasma IP10 levels were determined via quantitative ELISA. Differences between TLR9 1486CC and CT/TT genotypes with respect to (**a**) CD4 counts and (**b**) CD8 immune activation is shown. (**c**) Comparison of CD8 immune activation between TLR9 1486 CC, CT or TT genotypes. (**d**) Plasma IP10 levels between TLR9 1486CC or CT/TT genotype groups. One tailed student’s t-test or Kruskal-Wallis test was used for statistical analysis

Several other polymorphisms in the TLR9 region like 1486C/T in the TLR9 promoter have been shown to be associated with diseases like lupus [53] and rheumatoid arthritis [40]. However, there are no reports on the association of TLR9 1486C/T polymorphism with HIV infection or disease progression. We hence looked at the association of TLR9 1486C/T polymorphism with HIV clinical parameters in our cross sectional patient cohort. Interestingly, 1486C/T polymorphism was found to be in linkage disequilibrium with TLR9 1635A/G in our HIV patient population (D’ 0.943). As shown in Fig. 7a, presence of TLR9 1486CC genotype was associated with lower CD4 counts compared to 1486CT/TT, although the difference was not statistically significant. Similarly, CD8 T cell immune activation, a hallmark of HIV disease was also higher for the TLR9 1486CC group (Fig. 7b). Further stratification of data into 1486CC, CT or TT genotypes showed that CD8 immune activation was again highest for 1486CC followed by CT and least for the TT genotype (Fig. 7c). These HIV disease progression markers that were significantly different for the 1635A/G polymorphism only showed a trend but did not reach statistical significance for the 1486C/T analysis. However, the exception was plasma IP10 levels that were significantly higher (*p* = 0.031) for TLR9 1486CC compared to CT/TT genotypes (Fig. 7d). These data suggest a possible association of TLR9 1486C/T polymorphism with HIV induced CD4 T cell decline, T cell immune activation and increased plasma IP10 levels, although this association was weaker than that seen with 1635AA genotype.

### TLR9 1635AA combined with 1486CC genotype accounts for reduced CD4 counts, increased CD8 immune activation and higher plasma IP10 levels in HIV+ patients

**Fig. 8:**
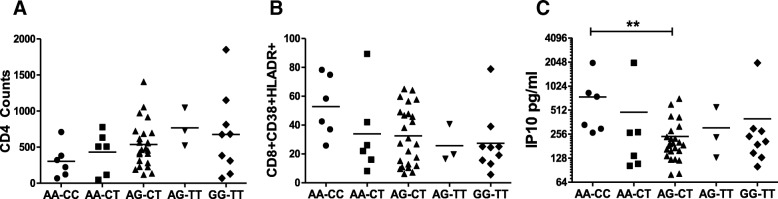
Combination of TLR9 1635AA-1486CC genotype is associated with lower CD4 counts, higher T cell immune activation and elevated IP10 levels in HIV infected patients. TLR9 1635 and 1486 polymorphisms were determined by PCR-RFLP analysis. Data was stratified based on both the 1635 and 1486 genotypes for each patient. Association of TLR9 1635 and 1486 genotype with (**a**) CD4 counts (**b**) CD8 immune activation and (**c**) plasma IP10 levels in HIV infected individuals is shown. Kruskal-Wallis test was used for statistical analysis (** *p* < 0.005)

Our data above demonstrated that in our patient population, both TLR9 1635A/G and 1486C/T polymorphisms were likely determinants of CD4 decline, T cell immune activation and elevated plasma IP10 levels. As 1486C/T polymorphism was found to be in linkage disequilibrium with TLR9 1635A/G, we investigated the combined effect of these polymorphisms on some key parameters of HIV disease. As demonstrated in Fig. 8a, the 1635AA genotype combined with 1486CC was associated with the lowest CD4 counts followed by AA-CT, AG-CT, AG-TT and GG-TT. This correlated inversely with CD8 immune activation with the highest immune activation seen for 1635AA-1486CC followed by AA-CT, AG-CT, AG-TT and GG-TT (Fig. 8b). Furthermore, levels of plasma IP10 followed the same pattern as CD8 immune activation with highest IP10 levels seen for the 1635AA-1486CC genotype which was significantly different from AG-CT genotype (*p* < 0.05) (Fig. 8c). Interestingly, in this stratification of data the AA-CT genotype was not different from AG-CT genotype suggesting a combined role of both 1635A and 1486C TLR9 polymorphisms in HIV mediated CD4 decline and immune activation.

## Discussion

HIV pathogenesis is a multifactorial and complex phenomenon involving multiple host and viral factors [54]. Chronic immune activation is a hallmark of HIV infection and correlates with CD4 loss and disease progression [1, 2]. The role of differential immune activation is also fundamental to pathogenic versus non-pathogenic SIV infections in non-human primates [55, 56]. Multiple hypotheses have been proposed for HIV associated immune activation [46] including microbial translocation and involvement of toll like receptors [5, 46]. Interestingly, HIV disease progression also correlates with viremia, supported by a direct correlation between immune activation and viremia [2], reduction in immune activation as a result of virus suppression by antiretroviral therapy [4, 10–12] and role of residual viremia in mediating immune activation in patients that fail to control virus replication [13].

The association between immune activation and viremia suggests that TLR family of innate sensors may be involved in this phenomenon [57]. In fact, previous studies have indicated that TLR7, that senses viral RNA and TLR9 that senses unmethylated CpG viral DNA may be involved in HIV mediated immune activation [14, 17]. The sensing of viral nucleic acids by plasmacytoid dendritic cells and subsequent Interferon-1 (IFN-1) production has been proposed as a mechanism behind this phenomenon [15]. A recent study by O’Brien et al shows that HIV envelope-CD4 interactions are key determinants of plasmacytoid dendritic cell stimulation and IFN production [21]. TLR9 can also be stimulated by CpG DNA from bacteria and incidentally microbial translocation as well as plasma levels of bacterial DNA are both increased in HIV patients and correlate with immune activation [6, 58]. Further support of a role of TLRs in HIV immune activation comes from studies showing that inhibitors of TLR7 and TLR9 signaling like chloroquine and hydroxychloroquine can inhibit HIV mediated immune activation [20, 59].

A growing number of studies have demonstrated an association of different SNPs in the TLR genes with susceptibility to infectious diseases [60]. As TLR9 is an important innate immune sensor, it is not surprising that polymorphisms within this gene have been shown to associate with infectious and inflammatory diseases including HIV-1 [33, 36, 37], SLE [39] and malaria [26, 61]. Specifically, the TLR9 1635A/G polymorphism (also referred to as 2848C/T in some studies) has been associated with thrombocytic thrombocytopenic purpura (TTP) [62], meningococcal meningitis [27], cytomegalovirus infection in fetuses and newborns [63], symptomatic malaria [26] and toxoplasmosis [64]. In the context of HIV, TLR9 1635A/G polymorphism has previously been shown to associate with HIV acquisition/infection [30, 32], disease progression [33, 34], CD4 counts [35, 36] and set point viremia [37]. Another polymorphism, 1486C/T in the promoter region has also been associated with pulmonary tuberculosis [41], gastric carcinoma [42] and SLE [53], although the role of this polymorphism in HIV has not been studied. Interestingly, we found that in our patient population that includes mostly hispanics, the 1635A/G and 1486C/T SNPs were in fact in linkage disequilibrium.

Our study is the first to demonstrate a clear association between TLR9 1635A/G polymorphism and HIV mediated immune activation in both CD4 and CD8 T cells. Furthermore, our study provides a mechanistic insight into how TLR9 1635A/G polymorphism may be affecting HIV disease outcome by influencing IP10 production and immune activation. In support of this hypothesis, in vitro TLR9 stimulation has been shown to mediate immune activation including upregulation of activation markers CD38 and HLA-DR, similar to what is seen in HIV infections [19]. Furthermore, TLR9 stimulation via CpG has been shown to induce IP10 production from monocytes, plasmacytoid dendritic cells and B cells [49, 50]. Interestingly, IP10 has been shown to be elevated in HIV infections [7, 47] and associated with CD4 decline [65], immune activation [66] and plasma viral load [65]. Recent studies have also shown IP10 to be a highly sensitive marker of viremia in acute HIV infections [52, 67]. Thus, an interconnection between HIV viremia, TLR9, IP10 and immune activation can be inferred both from our study and findings by others.

With regards to the molecular mechanism behind various SNPs affecting TLR9 expression/function, 1635A/G polymorphism is located in the coding region of the protein and introduces a silent mutation that does not change the amino acid sequence. We conducted protein expression studies in HeLa cells using an HA tagged TLR9 gene expressed via a CMV promoter that did not reveal significant differences in TLR9 protein expression between the 1635A or G variants (data not shown). On the other hand, studies by Tao et al suggest that TLR9 1486C allele may reduce TLR9 expression by affecting promoter activity [39]. Although in our sample set, TLR9 1635 A/G and 1486C/T were in linkage disequilibrium, the weak association of TLR9 1486C/T with CD4 decline compared to 1635A/G suggests a more complex role of these two SNPs in TLR9 expression and or function. The fact that in our patient population, these two SNPs were found to be in linkage disequilibrium indicates that perhaps 1635A/G and 1486C/T collectively affect TLR9 expression and/or function. In support of this, our analysis of the data after stratifying into haplotypes/diplotypes showed that 1635AA combined with 1486CC genotypes showed highest levels of plasma IP10 levels.

Our findings provide some new insights into HIV mediated immune activation and pathogenesis. Firstly, the association of TLR9 1635A/G polymorphism with CD4 levels in HIV patients is confirmed in our study. More importantly, we show here for the first time that TLR9 1635A/G polymorphism is associated with immune activation in both CD4 and CD8 T cells in HIV patients as well as plasma IP10 levels. Our findings suggest a possible mechanism behind the association of TLR9 1635A/G polymorphism and HIV pathogenesis by regulating immune activation. However, several questions remain unanswered like which cells produce IP10 and the role it plays in T cell activation if any. Whether plasmacytoid dendritic cells from TLR9 1635AA genotype are more prone to activation or produce higher levels of IP10 when stimulated with HIV remains to be seen.

Our study has obvious limitations including a cross sectional rather than longitudinal design and a small sample size. Our patients were at different disease stages and not all the patients were on HAART at the time of sample collection which can be confounding factors. Furthermore, different treatment regimens can also affect inflammatory markers. The statistical significance in our data set is limited except for certain parameters like T cell activation and IP10 levels. This might be due to the multifactorial nature of HIV disease. However, our findings fit into the larger narrative of HIV pathogenesis and role of TLR9 in this process. Further studies including longitudinal analysis will be needed to determine the role of TLR9 polymorphisms in HIV disease progression. Also, the molecular mechanism by which TLR9 1635A/G and 1486C/T polymorphisms affect TLR9 expression and/or function remains undetermined and open for further investigation.

## Conclusions

Our study provides some new insights into the mechanism of HIV mediated immune activation and pathogenesis. In a cohort of HIV infected patients, we found that TLR9 1635AA genotype was associated with lower CD4 counts and significantly higher immune activation in both CD4+ and CD8+ T cells along with higher plasma IP10 levels. Another TLR9 polymorphism, 1486C/T that is in linkage disequilibrium with 1635A/G was also weakly associated with lower CD4 counts, higher CD8 immune activation and higher IP10 levels. As dendritic cells respond to TLR9 stimulation via IP10 production, in context of HIV infection, TLR9 stimulation by viral CpG DNA may regulate immune activation and CD4 loss. Our data supports an association between TLR9 polymorphisms and HIV mediated immune activation.

## Abbreviations

AIDS: Acquired Immune Deficiency Syndrome
ART: Anti Retroviral Therapy
HIV: Human Immunodeficiency Virus
IFN: Interferon
LAL: Limulus Amebocyte Lysate
LPS: Lipopolysaccharide
PRRs: Pattern Recognition Receptors
TLR: Toll Like Receptor
